# Valgus-impacted subcapital neck of femur fractures: a systematic review, meta-analysis with cost analysis of fixation *in-situ* versus nonoperative management

**DOI:** 10.1177/11207000231210240

**Published:** 2023-11-21

**Authors:** Herv Vidakovic, David Kieser, Gary Hooper, Chris Frampton, Michael Wyatt

**Affiliations:** Department of Orthopaedic Surgery and Musculoskeletal Medicine, Christchurch, New Zealand

**Keywords:** NOF, neck of femur fracture, valgus-impacted, meta-analysis

## Abstract

**Background:**

The management of the valgus-impacted neck of femur fracture (AO/OTA 31-B1) remains contentious. The objective of this study was to determine whether operative intervention is cost-effective.

**Methods:**

We conducted a systematic review using electronic databases (Medline, Embase, Cochrane, Ebsco, Scholar) identifying studies published in the English language concerning valgus-impacted neck of femur fractures until June 2022. Additional studies were identified through hand searches of major orthopaedic journals, and bibliographies of major orthopaedic textbooks. MeSH terms (hip fracture and femoral neck fracture) and keywords (undisplaced, valgus-impacted, valgus, subcapital, Garden) connected by the Boolean operators “AND” and “OR” were used to identify studies. 2 reviewers independently extracted the data using standardised forms and recording spreadsheet. Methodological validity prior to inclusion in the review using standardized critical appraisal instruments from the Joanna Briggs Institute Meta-analysis of Statistics Assessment and Review Instrument. Meta-analysis was undertaken. Outcome measures were rate of displacement, avascular necrosis, non-union, mortality and requirement of further operative intervention. A cost utility analysis was then conducted to compare the 2 groups on the basis of the cost of initial treatment and the potential requirement of secondary intervention to hemiarthroplasty.

**Results:**

47 studies met the inclusion criteria. Meta-analysis data demonstrated a significant difference in the displacement rate of 22.8% and 2.8% between the nonoperative and internal fixation groups respectively (*p* = 0.05). The overall incidence of further operative intervention for each group was 23% and 10% respectively. There was no significant difference with respect to avascular necrosis, mortality or union rates. The cost utility analysis revealed nonoperative management to be approximately 60% more costly than initial internal fixation when the costs of subsequent surgery were included.

**Conclusions:**

This meta-analysis of the existing literature concludes that whilst nonoperative management is possible for valgus impacted neck of femur fractures, it is associated with higher complication rates and greater expense than management by internal fixation.

## Introduction

Neck of femur fractures (NOF#) represent 1 of the most common orthopaedic injuries requiring operative intervention with an estimated 1.5 million sustained worldwide annually.^1^ Valgus impacted subcapital NOF# (AO/OTA 31-B1) represent approximately 15–20% of these injuries.^2^ This fracture has classically been thought to have inherent stability due to its vector of impaction in the coronal plane.^2,3^ This has led some authors to recommend nonoperative management for this fracture pattern, ranging from prolonged bed rest to early weightbearing as tolerated.^2,4–8^ However, since Smith-Petersen first popularised fixation of valgus-impacted NOF# in 1931, operative management has become increasingly utilised.^9–11^

In support of this trend, multiple publications have reported a high complication rate associated with nonoperative management of this condition, particularly fracture displacement and symptomatic avascular necrosis of the femoral head necessitating conversion to hip arthroplasty.^5,12–17^ However, operative management has also been associated with a range of complications, including perioperative mortality, periprosthetic fracture, metalware failure and joint penetration.^18–22^ Thus some authors continue to advocate conservative management of this fracture.^2,23^ Unfortunately, there have been few systematic reviews and, to the best of our knowledge, no randomised control trials directly comparing current operative and non-operative management of this specific fracture pattern.^24,25^

Another facet worth understanding is the economic cost of this injury. Internationally, as our population continues to age and the annual rate of NOF# continue to rise, the economic burden of this injury needs to be addressed.^26–29^ While there are a number of studies reporting the cost effectiveness of various operative treatments for NOF#, there are none comparing conservative with operative fixation in-situ of valgus-impacted subcapital NOF#.^30–33^

The aim of this study is therefore to critically review the existing literature to firstly assess the risk profile of operative versus nonoperative management of valgus impacted subcapital NOF# in patients >60 years of age. Secondly to assess the failure rates of these 2 modalities as defined as conversion to hip arthroplasty. Lastly to compare the economic costs of these 2 approaches.

## Methods

### Search criteria

This analysis was conducted in accordance with the Preferred Reporting Items for Systematic Reviews and Meta-Analyses (PRISMA) and the Meta-analysis of Observational Studies in Epidemiology guidelines.^34^

The search strategy aimed to find both published and unpublished studies reporting on valgus impacted subcapital NOF#. A 3-step search strategy was utilised.

Firstly, a comprehensive search of all publications in the electronic databases up to June 2022 was independently conducted by two investigators (HV and DK) using Medline, Embase, Google Scholar and the Cochrane Library. The MeSH terms (hip fracture and femoral neck fracture) and keywords (undisplaced, valgus-impacted, valgus, subcapital, Garden) connected by the Boolean operators “AND” and “OR” were used to identify all possible studies. The identified articles were then reviewed for keywords contained in the title, abstract, and the index terms used to describe the study subject. Secondly, a search using all identified keywords and index terms was undertaken across all of the included databases. Finally, a manual search was conducted of the reference lists of identified articles and relevant reviews for additional studies.

### Inclusion criteria

All studies had to meet the following inclusion criteria: (1) studies reporting on the outcomes of undisplaced, valgus-impacted neck of femur fractures managed with either operative fixation *in-situ* (regardless of implant selection), or conservative management with permitted early weightbearing; (2) randomised control trials, non-randomised control trials, observational studies, or registry studies: (3) report on at least the primary outcome measure detailed below, ⩾2 secondary outcomes of interest; (4) minimum follow-up of 6 months; (5) studies including patients aged >60 years only; (6) studies published in the English language.

### Exclusion criteria

Studies were excluded if they had any of the following characteristics: (1) reviews, abstracts, editorials, expert opinions, or letters; (2) duplicate data; (3) biomechanical studies; (4) unclear methodology or ambiguous outcome reporting; (5) studies with overlapping data of displaced subcapital neck of femur fractures (6) primary treatment with arthroplasty.

### Article assessment

All papers selected for inclusion were assessed independently by 2 reviewers (HV and DK) for methodological validity prior to inclusion in the review using standardised critical appraisal instruments from the Joanna Briggs Institute Meta Analysis of Statistics Assessment and Review Instrument (JBI-MAStARI).^35,36^ Any disagreements that arose between the reviewers were resolved through discussion with a third reviewer (CF).

### Outcomes of interest

The primary outcome measure for this meta-analysis was fracture displacement in the conservatively managed group, and either fracture displacement or implant failure in the operatively managed group. Displacement was defined as any reported disimpaction of the femoral head from the femoral neck. Any reported progressive impaction was not considered as displacement. Implant failure was defined as perforation of the implant into the hip joint, implant cut out from the femoral head, or periprosthetic fracture. Prominent metalware was not considered as implant failure. Secondary outcome measures were; union, avascular necrosis (AVN) of the femoral head, mortality, length of hospital stay and the need for subsequent surgery for any cause in either treatment group.

The economic component of this review assessed the cost utility of treatment by utilising studies that have a published cost itemisation, and undertaken cost analyses of each treatment option as well as the cost of secondary intervention.^32,33^ The cost analysis was extrapolated for the outcomes found from the meta-analysis to determine the relative cost utility of each treatment option based on the sum total of attributable itemised costs for the primary intervention, and subsequently the costs incurred per group from need for subsequent surgery for fracture displacement with conversion to hemiarthroplasty.

### Data extraction

2 reviewers (HV and DK) independently extracted quantitative data from all eligible studies using a standardized data recording spreadsheet. Disagreements were resolved by discussion with a third reviewer (CF). The data of interest included the following categories: (1) study characteristics such as study type, year of publication, cohort size, age, follow-up duration and numbers lost to follow up; (2) treatment information including operative or conservative management with early weightbearing and hospital stay; (3) numbers of patients with the primary outcome of fracture displacement in the conservatively managed group and either displacement or implant failure in the operatively managed group; (4) numbers of patients with secondary outcomes including avascular necrosis, non-union, mortality, and secondary intervention with “revision” surgery for any cause in either treatment group. In addition, time to displacement, ongoing pain and postoperative infection were documented if reported.

### Statistical analysis

For each included study, means and confidence intervals (CIs) were calculated for incidence of outcomes, along with risk ratios (RR) and numbers needed to treat or harm (NNT/NNH). Heterogeneity across studies was assessed with use of both the chi-square (*χ*^2^) test and the *I*-squared (*I*^2^) test. Statistical heterogeneity was considered significant when *p* < 0.10 for the *χ*^2^ test or *I*^2^ > 50%. Random and fixed effects models were used to ensure that these studies represented a random sample of all potentially available studies. Sensitivity analysis was performed to test the strength and robustness of pooled results by sequential omission of individual studies. Publication bias was assessed using a funnel plot of the most frequently reported outcome. All reported p*-*values were 2-sided and *p* < 0.05 was regarded as statistically significant. Statistical analyses were conducted by RevMan 5.2.10 software (Cochrane Collaboration, UK). All numerical analyses were reported to 2 decimal points.

## Results

### Included studies

A total of 2349 studies were identified in the initial database search. After incorporation of the inclusion and exclusion criteria 46 studies met the required criteria (Table 1). This included 38 observational studies, 3 randomised control trials (RCTs), 4 non-randomised control trials and 1 registry study. The randomised control trials included in the meta-analysis were ones comparing different implants for fixation *in-situ*. There were no randomised control trials comparing conservative with operative management identified in the literature search. Of the 46 articles, 29 reported only on the outcomes of operative fixation *in-situ* and 12 reported only on conservative management. 5 studies directly compared patients treated operatively and conservatively, none of which stated randomisation as part of their study design.

**Table 1.** Studies identified.

Study	Type	Management	Mean Follow up	Cohort Size	Displacement/ Implant failure	Non-union	AVN	Revision Surgery	Mortality
Hansen^6^ (1978)	Obs	Conservative	6 months	42	8	0	3	10	16%
	RCT	Operative	2 years	27	0	0	6	4	22%
Rogmark^18^ (2009)	Obs	Operative	2.5 years	224	1	12	10	34	22%
Cserháti^16^ (1996)	Obs	Conservative	2 years	122	24	1	5	22	14%
		Operative	2 years	125	1	0	10	10	10%
Buord^15^ (2010)	Obs	Conservative	1.5 years	57	19	0	1	18	14%
Raaymakers^37^ (1991)	Obs	Conservative	3 years	170	24	0	9	21	16%
Jensen^8^ (1983)	Obs	Conservative	2 years	85	9	0	3	0	2%
Otremski^5^ (1990)	Obs	Conservative	3 years	123	11	0	9	0	UK
Tanaka^7^ (2002)	Obs	Conservative	20 months	38	23	15	0	0	UK
Raaymakers^2^ (2002)	Obs	Conservative	2 years	319	95	0	18	109	19%
Shuqiang^17^ (2006)	Obs	Conservative	1 year	115	48	0	0	48	UK
Bentley^38^ (1968)	Obs	Conservative	2 years	43	7	1	5	6	17%
		Operative	2 years	23	8	0	2	0	17%
Bjørgul^21^ (2007)	RCT	Operative	3 years	225	9	16	10	42	22%
Verheyen^12^ (2005)	Obs	Conservative	6 months	105	48	0	0	48	UK
Chen^39^ (2005)	Obs	Operative	2 years	40	2	2	4	6	8%
Chiu^22^ (1996)	Obs	Operative	2 years	318	9	15	18	35	21%
Conn^24^ (2004)	Obs	Operative	1 year	414	6	24	15	29	21%
Gjertsen^40^ (2011)	Reg	Operative	1 year	5205	436	0	0	436	UK
Lawrence^41^ (2011)	Obs	Operative	1 year	211	0	32	16	UK	UK
Lee^42^ (2004)	Obs	Operative	2 years	104	0	6	4	2	7%
Lee^43^ (2008)	Obs	Operative	1 year	102	3	1	10	6	12%
Murphy^44^ (2013)	Obs	Operative	3 years	358	25	20	9	53	UK
Nilsson^45^ (1988)	Obs	Operative	5 years	129	8	8	10	8	21%
Yoon^46^ (2012)	Obs	Operative	2 years	31	0	2	3	7	UK
Strömqvist^47^ (1992)	Obs	Operative	2 years	175	0	5	4	6	29%
Strömqvist^48^ (1987)	Obs	Operative	2 years	85	0	1	2	3	25%
	Obs	Operative	2 years	100	4	4	3	7	27%
Parker^26^ (2008)	Obs	Operative	1 year	346	2	38	13	50	19%
Bunata^49^ (1973)	Obs	Operative	2 years	53	0	0	0	0	UK
		Conservative	2 years	60	7	7	0	0	UK
Flatmark^4^ (1962)	Obs	Conservative	1 year	51	4	1	10	0	27%
Doran^50^ (1989)	Obs	Operative	2 years	58	2	0	4	21	UK
Barnes^51^ (1976)	Obs	Operative	3 years	295	0	2	9	0	5%
Lagerby^52^ (1998)	RCT	Operative	1 year	75	0	1	3	4	20%
Cobb^53^ (1986)	Obs	Operative	2 years	43	0	0	3	4	9%
Bedat^14^ (1997)	Obs	Conservative	1 year	124	52	1	4	49	5%
		Operative	1 year	54	0	2	1	0	0%
Parker^54^ (2013)	Obs	Operative	1 year	112	0	3	5	11	17%
Zlowodzki^19^ (2005)	Obs	Operative	6 months	57	11	UK	UK	UK	UK
Palm^55^ (2009)	Obs	Operative	1 year	113	18	4	4	31	UK
Hilleboe^56^ (1970)	Obs	Conservative	1 year	37	4	0	3	4	UK
Levi^57^ (1995)	Obs	Operative	1 year	252	UK	25	0	UK	10%
Tidermark^58^ (2002)	Obs	Operative	2 years	24	1	UK	1	2	33%
Hui^59^ (1994)	Obs	Operative	6 months	57	10	0	1	11	21%
Lapidus^60^ (2013)	Obs	Operative	3.5 years	382	7	25	20	72	21%
Stappaerts^61^ (1987)	Obs	Operative	3 years	33	0	1	2	UK	UK
Clement^62^ (2013)	Obs	Operative	1 year	120	21	UK	UK	21	19%
Shin^63^ (2021)	Obs	Conservative	Unknown	55	15	15	0	UK	UK
Goodnough^64^ (2021)	Obs	Operative	13 months	97	15			15	1.5%
Goodnough^64^ (2021)	Obs	Conservative	6.5 months	28	10	10	0		9.1%

#### AVN, avascular necrosis; UK, unknown

All studies were published between the years of 1962 and 2021. Studies reporting on conservative management outcomes were published in years ranging from 1962 to 2006 and operative management from 1968 to 2021. The cohort size of each study ranged from 24 to 5405. Mean follow-up ranged from 6 months to 5 years (Table 1).

### Outcomes

#### Displacement

44 of the included studies reported on displacement. Pooled analysis demonstrated a significantly lower mean displacement rate of 2.88% in the operatively managed group (CI, 1.58– 4.56) compared to 22.88% in the conservatively managed group (CI, 16.15–30.40) (*p* = 0.05). This equates to a risk ratio of 0.13 and a number needed to treat of 5 (Table 2).

**Table 2.** Pooled analysis of outcomes of conservative versus operative treatments.

		Mean %	lower 95	upper 95	Difference	NNT/NNH	Risk Ratio	*p*-value
AVN	Conservative	4.45	2.94	6.27	0.30	331	1.07	0.90
	Operative	4.76	2.87	7.08				
Mortality	Conservative	11.82	7.56	16.88	4.06	25	1.34	0.76
	Operative	15.88	12.51	19.56				
Displacement	Conservative	24.70	31.46	18.52	–21.55	–5	0.13	0.04
	Operative	3.15	1.76	4.92				
Revision	Conservative	23.20	14.63	33.06	–13.47	–7	0.42	0.49
	Operative	9.73	7.50	12.22				
Nonunion	Conservative	2.30	0.60	5.06	1.15	87	1.50	0.28
	Operative	3.45	1.79	5.61				

NNT/NNH, numbers needed to treat or harm; AVN, avascular necrosis.

**Figure 1.**
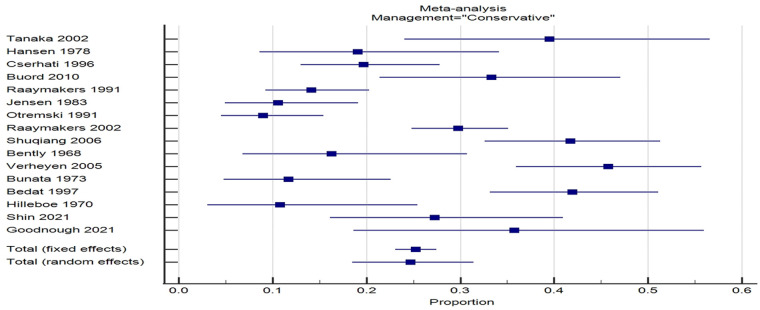
Forrest plot of studies reporting fracture displacement after initial nonoperative management.

**Figure 2.**
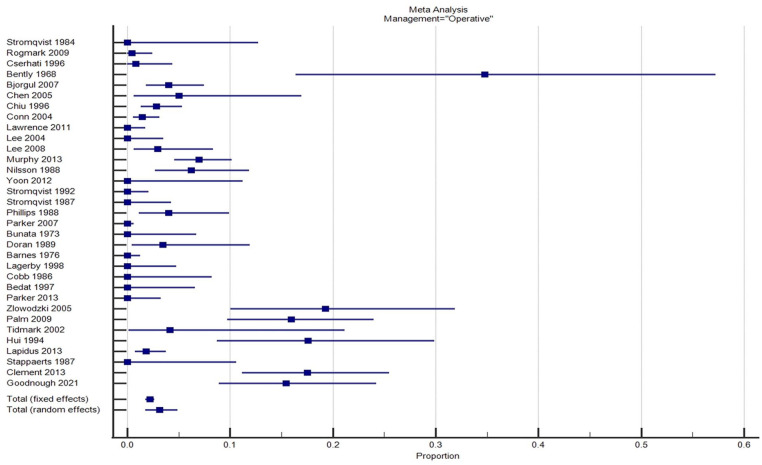
Forrest plot of studies reporting fracture displacement after initial operative management.

Significant heterogeneity existed between studies reporting on both types of management with respect to displacement rates with *I*^2^ of 89% for each group (Figures 1 and 2). However, variability between studies was less marked with operative fixation *in-situ* compared to conservative management suggesting a more predictable outcome in respect to fracture displacement (Figure 2).

#### Union

46 studies reported on the union rate. No significant difference was found between the 2 groups; 84% (CI, 80.4–87.6) operatively and 77% (CI, 67.95–85.25) nonoperatively (*p* = 0.95).

#### Avascular necrosis (AVN)

40 studies reported on the occurrence of AVN of the femoral head. Pooled estimates revealed a similar AVN rate of 4.76% (CI, 2.87–7.08) in the operative group and 5.1% (CI, 3.6–6.85) in the conservative group (*p* = 0.89).

#### Revision surgery

38 of the included studies reported on subsequent revision surgery for any cause following initial fixation *in-situ* or conservative management. The proportion of patients requiring subsequent surgery was 9.55% (CI, 7.3–12.06) in the operative group and 23.2% (CI, 14.63–33.06) in the conservative group. This was not found to be statistically significant (*p* = 0.48). There was inconsistent reporting amongst studies on the various indications for revision surgery, therefore further subgroup analysis was not possible. There was significant heterogeneity found between studies for both groups, with an *I*^2^ of 90% and 93% for operative and nonoperative groups respectively.

#### Mortality

29 of the included studies reported on mortality rate. The mean 1-year mortality rate was 16.79% (CI, 13.53–20.32) in the operative group and 12.51% (CI, 7.98–17.87) in the conservatively managed group. This was not statistically significant (*p* = 0.76).

#### Length of hospital stay

12 studies reported on the length of hospital stay, 5 of which reported on hospital stay for those patients managed nonoperatively. A significant difference with respect to mean length of hospital stay between the 2 groups was found, with an average hospital stay of 22 days in the nonoperative group and 12 days in the operative group (*p* < 0.01).

#### Cost analysis

An itemised cost analysis was carried out to compare the two groups on anticipated average costs of in hospital management. The published itemised costs (in Euro) were attained from a paper by Frihagen and colleagues in 2010 and applied to each group to calculate the mean total cost per patient based on treatments received and mean length of stay from the pooled estimates (Table 3). Further calculations were carried out on the cost per group to treat of the complication of fracture displacement with the assumption that they would require a subsequent hemiarthroplasty.

**Table 3.** Cost-analysis of operative versus operative management of valgus impacted neck of femur fractures.

Initial costs and follow-up costs	Cost (E)	Units	Conservative	Operative	Secondary intervention (Conservative)	Secondary intervention (Operative)
			*Cost*			
***Transportation***						
Ambulance	€ 222.00	Transfer	€ 222.00	€ 222.00	€ 222.00	€ 222.00
Emergency Department assessment	€ 356.00	Presentation	€ 356.00	€ 356.00	€ 356.00	€ 356.00
***Equipment (in surgery)***						
Internal fixation	€ 310.00	Operation	€ 0.00	€ 310.00	€ 0.00	€ 0.00
Hemiarthroplasty	€ 1,282.00	Operation	€ 0.00	€ 0.00	€ 1282.00	€ 1282.00
Removal of screws	€ 171.00	Operation	€ 0.00	€ 0.00	€ 0.00	€ 171.00
Bipolar hemi-cup (Hastings)	€ 652.00	Operation	€ 0.00	€ 0.00	€ 652.00	€ 652.00
***Anesthesia costs***						
Fixed anaesthesia costs	€ 87.00	Operation	€ 0.00	€ 87.00	€ 87.00	€ 87.00
Variable anaesthesia costs	€ 63.00	Hour	€ 0.00	€ 63.00	€ 126.00	€ 126.00
***Personnel costs (in surgery)***						
Orthopedic surgeon	€ 51.00	Hour	€ 0.00	€ 51.00	€ 102.00	€ 102.00
Orthopedic registrar	€ 39.00	Hour	€ 0.00	€ 39.00	€ 78.00	€ 78.00
Anaesthetic registrar	€ 39.00	Hour	€ 0.00	€ 39.00	€ 78.00	€ 78.00
Theatre nurse	€ 27.00	Hour	€ 0.00	€ 54.00	€ 54.00	€ 54.00
Anaesthetic technician	€ 27.00	Hour	€ 0.00	€ 27.00	€ 54.00	€ 54.00
Postoperative costs	€ 69.00	Hour	€ 0.00	€ 138.00	€ 138.00	€ 138.00
***Inpatient days***						
Overhead costs	€ 378.00	Day	€ 8316.00	€ 4536.00	€ 1890.00	€ 1890.00
Direct attention	€ 201.00	Day	€ 4422.00	€ 2412.00	€ 1005.00	€ 1005.00
Common costs	€ 250.00	Day	€ 5,500.00	€ 3,000.00	€ 1,250.00	€ 1,250.00
***Medication/blood transfusion***						
Cefalotin (Keflin)	€ 6.00	Dose	€ 0.00	€ 18.00	€ 18.00	€ 18.00
Dalteparin, LMWH (Fragmin)	€ 1.00	Dose	€ 42.00	€ 42.00	€ 42.00	€ 42.00
Oxycodone (Oxycontin)	€ 1.00	Dose	€ 14.00	€ 14.00	€ 14.00	€ 14.00
Codein/paracetamol (Pinex Forte)	€ 0.20	Dose	€ 33.60	€ 33.60	€ 33.60	€ 33.60
Lactulose	€ 0.10	Dose	€ 8.40	€ 8.40	€ 8.40	€ 8.40
Paracetamol	€ 0.10	Dose	€ 16.80	€ 16.80	€ 16.80	€ 16.80
Blood SAG	€ 143.00	units	€ 0.00	€ 0.00	€ 0.00	€ 0.00
***Radiology***						
Radiology image (x-ray)	€ 56.00	Set	€ 336.00	€ 392.00	€ 224.00	€ 224.00
***Laboratory***						
All labs	€ 87.00	Number	€ 87.00	€ 174.00	€ 174.00	€ 174.00
***Other post op contact***						
Outpatient clinic	€ 173.00	Visit	€ 865.00	€ 865.00	€ 865.00	€ 865.00
Telephone contacts	€ 19.00	Call	€ 95.00	€ 95.00	€ 95.00	€ 95.00
Physiotherapy visit	€ 25.00	Hours	€ 150.00	€ 150.00	€ 150.00	€ 150.00
					**Proportion revised in 1 Year**	
					**0.232**	**0.0955**
					**Initial conservative**	**Initial operative**
				**Total cost**	**20,463.80**	**13,142.80**
				**Total cost + cost for revision**	**22,588.41**	**14,033.70**

This cost analysis found a mean total cost per patient of €20,463.80 for those treated nonoperatively and €13,142.80 for those treated with fixation *in-situ*. A subsequent hemiarthroplasty for fracture displacement was factored in for each group, the cost per patient rose to €22,478.52 for those initially treated nonoperatively and €13,404.01 for those who underwent fixation *in-situ* (Table 3).

The principal contributor to cost was the direct and indirect costs of hospital stay, with the costs of surgery being a comparatively small 1-off cost, nearly equivalent to a single day in hospital.

## Discussion

With health projections revealing our ageing population and increasing numbers of NOF# it is essential to determine the optimal management of valgus impacted subcapital neck of femur fractures. Currently debate remains within the literature as to whether operative or nonoperative management of this condition is optimal

In this systematic review and meta-analysis, the pooled data demonstrates that valgus-impacted NOF# have an 8-fold higher rate of displacement (22.88 vs. 2.88 *p* = 0.05) if treated non-operatively, but a number needed to treat to prevent displacement of 5. Thus, only considering displacement, one might suggest that 80% of patients who undergo fixation *in-situ* undergo an unnecessary operation. However, due to this fracture being intracapsular displacement not only carries the risk of symptomatic mal-union or non-union, but also the risk of AVN and symptomatic femoral head collapse necessitating hip arthroplasty.

Interestingly, the difference in displacement rate is demonstrated to be statistically significant, but its clinical significance remains dependent on how the risks of subsequent surgery are perceived. Subjectively most surgeons would consider fixation *in-situ* to be a relatively benign procedure with reliable outcomes in comparison to hip arthroplasty. Thus, most contemporary surgeons prefer to fix these fractures *in-situ* to ensure retention of the patient’s native femoral head rather than risk fracture displacement and subsequent hip arthroplasty. However, the contemporary practice is called into question by Støen et al.,^65^ who demonstrated in an RCT that the clinical and functional outcomes of internal fixation and hemiarthroplasty are comparable at 6 years post displaced NOF#.^65^

Certainly, the variability in displacement rate seen between the studies reporting on the outcomes of conservative management are far more marked than with fixation *in-situ* suggesting that conservative management offers a less predictable treatment outcome.

Apart from displacement, the union rates, AVN, revision surgery and mortality rates between the groups were not significantly different. It should be noted the difference in rate of revision surgery between the two groups was less marked than that of displacement. The precise indications for revision surgery were reported inconsistently amongst the analysed studies, and therefore, a more detailed analysis was not possible. We assume that the majority of the subsequent surgery undertaken in the conservative group was for displacement, which based on contemporary practice would be to proceed to arthroplasty rather than reduction and fixation. In the fixation group, some studies listed a range of indications for revision surgery including, screw migration into the joint, fracture displacement, periprosthetic fracture, subcutaneous metalware irritation, deep infection, and avascular necrosis. Each of these would require a different management approach, and may account for the marked heterogeneity between studies in outcome reporting. This also makes it difficult to account for in a cost analysis, as a subgroup analysis was not possible for the revision surgery outcome.

The most notable secondary finding was that the nonoperatively managed group had a significantly longer mean length of hospital stay. This would imply a longer period of being incapacitated and independent immobile, but curiously, this implied potential for prolonged immobility associated with nonoperative management does not appear to be associated with a higher mortality rate in this meta-analysis.

The prolonged hospital stay of nonoperatively managed NOF# has major implications at a population level with respect to the utilisation and allocation of resources. Given the ageing population, increasing demand for hospital beds and shortage of healthcare resources it would be untenable to advocate for conservative management if the difference in length of stay is considered. This is particularly true in developed nations where the costs of hospital stay often outweigh the itemized costs of a surgical procedure.

The cost analysis conducted in this meta-analysis applied internationally accepted itemised costs to each treatment group. This demonstrates that the length of hospital stay represents the largest financial component, with fixation *in-situ* amounting to little more than the equivalent of 1 day in hospital. Although the exact costs of treatment may vary between countries and institutions, the proportional difference in treatment cost between conservative and operative management is important. This cost analysis supports operative management of valgus-impacted NOF# even if the clinical risk profile is to be rationalized. Given the ever-rising costs associated with an ageing population there is a need to account for providing cost-effective healthcare and demonstrate fiscal responsibility at the level of a clinician.

This study is largely limited by paucity of high-level evidence, including the absence of randomised control trials. In addition the vast heterogeneity of the small number of comparative trials limit this meta-analysis. Similarly, the large chronological spread of studies presented in this meta-analysis may introduce bias with respect to the variance in length of hospital stay, with current practice promoting early discharge planning with multi-disciplinary inputs.

Furthermore, this study is unable to illicit pain scores and functional outcome measures or assess individual patient radiographs to ensure those fractures that displaced were indeed valgus impacted without a significant posterior angulation. This study also fails to assess the optimal fixation method as all fixation types were grouped into the same category of fixation *in-situ.*

A further limitation is that we were only able to assess the inpatient aspects to treatment. There is very little literature to demonstrate what level of care patients with a valgus-impacted NOF# require after hospital discharge and whether or not fixation has any impact on this. We are also unable to account for the wider societal non-financial costs with either treatment option.

These limitations highlight the low quality of evidence on which the current standard of practice is based and emphasise the importance of a well-designed randomised control trial to assess the optimal management of this common injury.

Despite these limitations, this study offers the highest current level of evidence for the management of valgus impacted subcapital NOF# and concludes that fixation *in-situ* is superior to conservative management with a lower rate of fracture displacement, a shorter length of hospital stay and a lower total projected cost of treatment.
